# Soil pH Determines the Spatial Distribution, Assembly Processes, and Co-existence Networks of Microeukaryotic Community in Wheat Fields of the North China Plain

**DOI:** 10.3389/fmicb.2022.911116

**Published:** 2022-07-25

**Authors:** Yu Shi, Mengwei Xu, Yige Zhao, Liang Cheng, Haiyan Chu

**Affiliations:** ^1^State Key Laboratory of Crop Stress Adaptation and Improvement, School of Life Sciences, Henan University, Kaifeng, China; ^2^State Key Laboratory of Soil and Sustainable Agriculture, Institute of Soil Science, Chinese Academy of Sciences, Nanjing, China; ^3^University of Chinese Academy of Sciences, Beijing, China

**Keywords:** microeukaryote, soil pH, community assembly, co-occurrence network, North China Plain, rhizosphere

## Abstract

Soil microeukaryotes play a pivotal role in soil nutrient cycling and crop growth in agroecosystems. However, knowledge of microeukaryotic community distribution patterns, assembly processes, and co-existence networks is greatly limited. Here, microbial eukaryotes in bulk and rhizosphere soils of the North China Plain were investigated. The results showed that soil pH was the driving factor for the microeukaryotic community composition in the bulk and rhizosphere soils. The soil microeukaryotic community could significantly differ between alkaline and acidic soils. The results indicated that the soil pH had a stronger effect than niche differences on community composition. Partial Mantel tests showed that soil pH and spatial distance had similar effects on the microeukaryotic community composition in the bulk soil. However, in the rhizosphere soil, spatial distance had a stronger effect than soil pH. Infer Community Assembly Mechanisms by Phylogenetic bin-based null model (iCAMP) analysis revealed that drift was the most important process driving microeukaryotic community assembly, with an average relative importance of 37.4–71.1%. Dispersal limitation displayed slightly greater importance in alkaline rhizosphere than in alkaline bulk soils. Meanwhile, the opposite trend was observed in acidic soils. In addition, the contribution of each assembly process to each iCAMP lineage “bin” varied according to the acidic or alkaline conditions of the soil and the niche environment. High proportions of positive links were found within the four ecological networks. Alkaline soil networks, especially the alkaline bulk soil network, showed greater complexity than the acidic soil networks. Natural connectivity analysis revealed that the rhizosphere community had a greater stability than the bulk soil community in alkaline soil. This study provides a foundation for understanding the potential roles of microbial eukaryotes in agricultural soil ecosystem functioning.

## Introduction

Soil microbial eukaryotes (microeukaryotes) including fungi and protists are key soil residents that play a pivotal role in terrestrial ecosystem functioning (Fierer, 2017; Delgado-Baquerizo et al., 2018, 2020). Fungi, for example, are the central players in soil nutrient cycling, dead plant decomposition and disease mediation (Tedersoo et al., 2014; Soudzilovskaia et al., 2019; Liu et al., 2021; Zhang et al., 2022). Meanwhile, protists, which comprise an important component of the soil microbiome, play a critical role in top-down interactions and soil food webs (Oliverio et al., 2020; Xiong et al., 2020; Aslani et al., 2021). Due to the critical role of microbial eukaryotes in ecological service, it is necessary to understand the diversity, assembly process, and co-existence patterns of microeukaryotic communities in various ecosystems (Crowther et al., 2019).

Understanding the factors driving microbial diversity and distribution is a core area of research in microbial ecology (Fierer and Jackson, 2006; Martiny et al., 2006). Soil pH, which regulates the soil capacity for storing and supplying nutrients (Slessarev et al., 2016), has a fundamental influence on microbial distribution patterns (Fierer, 2017; Jiao and Lu, 2020). However, there are few available reports on how pH affects soil microeukaryotes. Recently, Aslani et al. (2021) found that soil pH was the primary determinant of eukaryotic microbial community distribution on a global scale. However, most studies are from natural ecosystems (Fierer, 2017), such as forest (Yang et al., 2019) and grassland ecosystem (Oliverio et al., 2020), the effect of soil pH on microeukaryotes in agroecosystems is understudied (Shi et al., 2019a). Moreover, rhizosphere, a hotspot for beneficial interactions between plant roots and microbes (de Vries et al., 2020; Li et al., 2021), also harbors various microeukaryotes. However, our understanding of distribution patterns of microeukaryotes in rhizosphere is still limited in agricultural ecosystem (Pineda et al., 2017).

Several studies have reported that both deterministic (e.g., soil) and stochastic processes (e.g., drift) play important roles in soil microbial distribution (Martiny et al., 2006; Stegen et al., 2013; Dini-Andreote et al., 2015; Shi et al., 2018). These deterministic and stochastic processes entail five main scenarios. Dispersal limitation (DL), drift (DR), and homogenous dispersal (HD) are defined as stochastic process, while heterogeneous selection (HeS) and homogenous selection (HoS) are deterministic processes. These five scenarios have been carefully described by Shi et al. (2019a). Briefly, HeS refers to environments that are highly spatially heterogeneous (Vellend, 2010); HoS refers to situations with spatially homogeneous environments (Shi et al., 2019a); HD describes high rates of dispersal between communities (Shi et al., 2019a); DL refers to spatial isolation (Whitaker et al., 2003; Zhou et al., 2008); and DR describes situations of ecological drift (Dini-Andreote et al., 2015; Feng Y. et al., 2018).

The five scenarios have been well-described in various habitats (Zhou et al., 2014; Feng M. M. et al., 2018; Jiao and Lu, 2020). For example, Feng Y. et al. (2018) revealed the relative role of these five processes in shaping soil microbial communities in long term fertilization fields. More recently, Aslani et al. (2021) suggested that drift is a dominant ecological process shaping soil eukaryotic community assembly on the global scale. However, their findings should be confirmed by further studies. Based on these findings, in this study, it was hypothesized that drift may be the dominant factor driving microeukaryotic community assembly in agricultural ecosystems (Orrock and Watling, 2010; Powell et al., 2015; Giner et al., 2018; Fodelianakis et al., 2021).

The tremendous numbers of microorganisms living in the soil are not independent, but form ecological networks involving mutualism, commensalism, amensalism, competitive parasitism, and predative relationships (Faust and Raes, 2012; Cardinale et al., 2015; Zhang B. et al., 2018; Ma et al., 2020; Shi et al., 2020). Microbial co-existence patterns involving prokaryotes, protists, and fungi have been well-described in marine (Lima-Mendez et al., 2015), forest (Ma et al., 2016), grassland (Shi et al., 2019b), and crop ecosystems (Xiong et al., 2019). Faust et al. (2015) revealed microbial association networks from 20 different cross-biome 16S rDNA sequencing datasets and observed that the tundra network contained a node representing pH. In the North China Plain, Shi et al. (2020) built a large-scale co- existence network of fungal and bacterial taxa using 243 soil samples, and they found the importance of the abundance of network hubs for soil functioning in wheat field systems.

Previous studies have revealed that microbial associations within biological community networks are critical for their stability (Neutel et al., 2002; Coux et al., 2016). Co-existence network approaches have been increasingly applied to reveal the stability of the association relationships among microbial individuals (Fan et al., 2018; Wu et al., 2021). Network robustness, calculated by the degree of natural connectivity through “attacking” (randomly removing) the edges and nodes within the network (Albert et al., 2000; Peng and Wu, 2016), is a method that is frequently being used to reflect network stability (Fan et al., 2018; Shi et al., 2019b; Wu et al., 2021). A greater network robustness indicates a more stable community, while a lower robustness reflects an unstable community. It is generally considered that soil microeukaryotes also form ecological associations. However, far fewer studies have specifically investigated co-existence patterns for these critical functioning players and their stability in agroecosystems (Zhang W. J. et al., 2018).

The North China Plain is the most important food-producing area in Asia, providing over 50% of China’s total cereal production (Piao et al., 2010; Jeong et al., 2014). Previously, researchers revealed the distribution patterns of soil bacteria and fungi in this region (Shi et al., 2018, 2020), and found the driving effect of soil pH. Additionally, microbial assembly processes and co-existence patterns were investigated across the North China Plain (Shi et al., 2018, 2020). In particular, deterministic processes were found to dominate at a broad scale (Shi et al., 2018), and the abundance of keystone species within the soil microbial networks showed high soil functional potential in this region (Shi et al., 2020). Moreover, the wheat rhizosphere was found to display a less complex but more stable microbial association network than the bulk soil (Fan et al., 2018).

In the present study, to investigate soil microeukaryotic community distribution patterns, assembly processes and co-existence networks, 20 bulk soil samples and 20 rhizosphere soil samples were collected across four sites in the North China Plain. It was hypothesized that soil pH could be the main driver shaping the bulk and rhizosphere soil microeukaryotic communities, and that drift would play an important role in determining microeukaryotic community assembly.

## Materials and Methods

### Site Description and Sample Collection

To survey the assembly processes and co-existence patterns of the microeukaryotic communities, 20 bulk soil and 20 rhizosphere soil samples in wheat fields were collected across four sites in the North China Plain in late April 2018. To ensure that all quadrats in each site have similar soil pH values in this study, we chose four typical sites which could represent acidic and alkaline soils according to Shi et al.’s (2018) study. For example, in a site, all the rhizosphere and bulk soils are alkaline in the five plots, while in another site, all the soils are acidic. The four sites were in Daming county (DM), Sheqi county (SQ), Taihe county (TH), and Tengzhou county (TZ). Each site had dimensions of 10 km by 10 km (100 km^2^) (Supplementary Table 1). Within each site, there were five plots (four plots are from the four corners and one plot in the center; Supplementary Figure 1), with the plots being at least 6 km apart from any other plot. The topography of sampling area is flat, and the altitude of sampling sites is below 50 m above sea level. The sampling region has a warm temperate monsoon climate, with an average annual temperature of 8–15°C and the average annual precipitation of 500–1,000 mm. The soils of sampling sites were classified as Ochric Aquic Cambosols (Chinese soil taxonomy) in our study (Zhu et al., 2005).

At each plot, groups of wheat plants (with 6–8 plants in each group) were removed to collect the rhizosphere soil (Donn et al., 2015; Fan et al., 2017). To obtain the rhizosphere soil, the plants were first lightly shaken and then the tightly bonded soil that remained attached the root surface was collected. Next to each group of plants (∼25 cm), from an area without plants, 3–5 cores of topsoil (0–15 cm) were collected and mixed by drill as bulk soil. Finally, five bulk soil and five rhizosphere soil samples were obtained for each site (a total of four sites were surveyed). All collected soil samples were immediately shipped to the laboratory in a cooler at 4°C. To remove the visible roots, residues and stones, the soils were sieved using 2 mm mesh. The sample was then divided into two parts: one part was stored at 4°C for physicochemical analysis and the other was stored at 20°C for DNA extraction.

### Soil Physicochemical Analyses and DNA Extraction

To measure soil pH, fresh soil with a soil-to-water ratio of 1:5 was tested using a pH monitor (Thermo Orion-868, Boston, MA, United States). The soil moisture content of each sample was determined gravimetrically after oven-drying at 105°C for 16 h. A total of 0.5 g of fresh soil was used for DNA extraction. The soil DNA was extracted using a Power Soil DNA kit (MO BIO, Carlsbad, CA, United States) and purified with an Ultra Clean 15 DNA purification kit (MO BIO) following the manufacturer’s instructions. The soil DNA was then stored at −40°C.

### Polymerase Chain Reaction Amplification and High-Throughput Sequencing

The primers SSU0817F (5′-TTAGCATGGAATAATRRAATAG GA-3′) and 1196R (5′-TCTGGACCTGGTGAGTTTCC-3′) (Rousk et al., 2010) were used to target and amplify the microbial eukaryotic 18S rRNA V5–V7 region in each sample. The polymerase chain reaction (PCR) products were obtained under the following conditions: 94°C for 5 min, followed by 35 cycles of 94°C for 30 s, 50°C for 30 s, and 72°C for 30 s. The PCR products were sequenced using the Illumina MiSeq PE 250 platform. High-throughput data from this analysis were submitted to the National Center for Biotechnology Information (NCBI) Sequence Read Archive (SRA) under accession number SRP347607.

### Sequence Data Analysis

Raw data sequences were processed and analysed using QIIME 2 (version: 2019.7) following the workflow at https://qiime2.org (Bolyen et al., 2019). Briefly, to obtain the amplified sequence variants (ASVs), Deblur was used to perform the quality control of the raw sequencing data (Amir et al., 2017). Low quality regions of the sequences were removed according to the sequence quality plot (each sequence was truncated at 120 bp). To identify and filter chimeras, vsearch was used to perform *de novo* chimera filtering. Based on the Sklearn-based taxonomy classifier, taxonomy assignment was performed using the dynamic Unite database from 10.10.2017.^1^ To rarefy the sequence number, 10,539 high-quality sequences were randomly selected for each sample.

### Analysis of Soil Microeukaryotic Community Assembly Processes

To reveal the soil microeukaryotic community assembly processes, iCAMP was selected (Ning et al., 2020). Using this approach (which can quantitatively explain community assembly mechanisms through phylogenetic bin based null model analysis), five assembly processes could be examined: DR, HD, DL, HeS, and HoS. In brief, the five processes could be observed via three major steps. The first step is phylogenetic binning. The second step is bin-based null model analysis, which partitions deterministic and stochastic processes into HoS, HeS, HD, DL, and DR. The final step is then to integrate the results for different bins to assess the relative importance of each process to them (Ning et al., 2020). In this study, 43 microeukaryotic bins were obtained. The confidence index was used for null model significance testing.

### Co-existence Analysis

The Sparse Correlations for Compositional data (SparCC) package was used to construct the microeukaryotic co-existence network following the procedure carefully described by Weiss et al. (2016). Before network construction, the ASVs table was filtered to improve the reliability of the networks. Four networks were constructed corresponding to the acidic rhizosphere soil network (AcRN), acidic bulk soil network (AcBN), alkaline rhizosphere soil network (AlRN), and alkaline bulk soil network (AlBN). Firstly, singletons were removed and only ASVs with an abundance of more than 0.01% of all samples in each group were retained. Finally, 295, 297, 301, and 379 ASVs were retained in AcRN, AcBN, AlRN, and AlBN, respectively. Then, the filtered ASVs tables were selected to construct the networks. All network topological features were quantified using the R ‘‘igraph’’ package^2^ and network visualizations were generated using Gephi.^3^

To investigate the microeukaryotic community stability, the robustness test was selected. To test the robustness of the networks, the natural connectivity was estimated by “attacking” the nodes (May, 1973) or edges (Jordan, 2009) of the SparCC network. To identify the network hubs, module hubs and connectors of each network, the z and c scores of each node within each network were calculated. Based on the threshold values of the z score (within-module degree) and the c score (participation coefficient) of nodes: nodes with a z score > 2.5 and c score > 0.6 were classified as network hubs; nodes with a z score > 2.5 and c score < 0.6 were classified as module hubs; nodes with a z score < 2.5 and c score < 0.6 were classified as connectors; and nodes with a z score < 2.5 and c score < 0.6 were classified as peripherals. The role of the network hubs, module hubs and connectors within networks have been carefully described by Shi et al. (2019b).

## Results

### Soil Microeukaryotic Communities and Diversity

**FIGURE 1:**
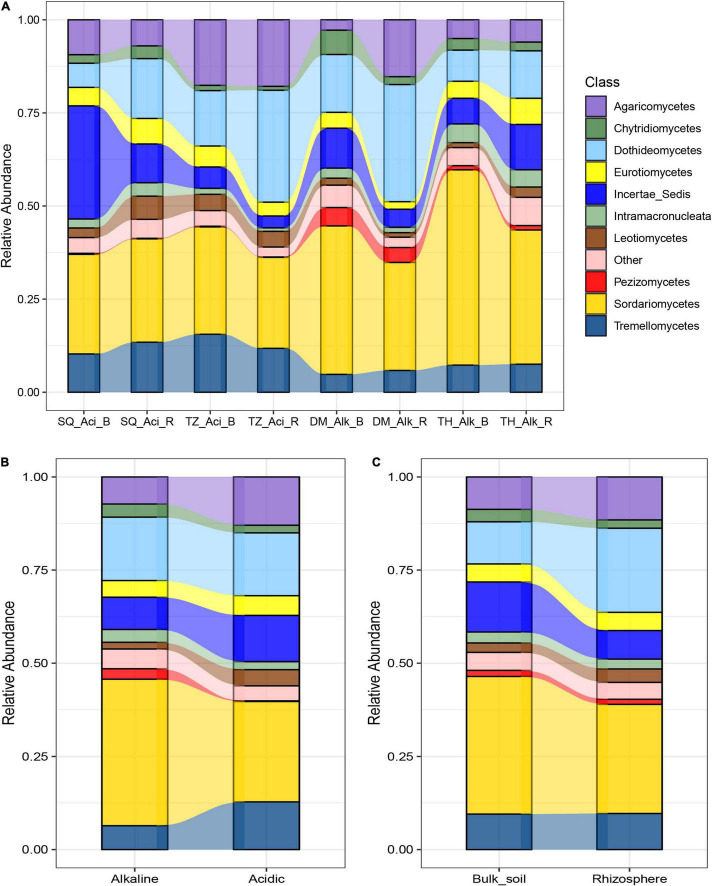
Relative abundance of the dominant soil microeukaryotic groups in alkaline (Alk), acidic (Aci), bulk (B), and rhizosphere (R) soils from four sites in the North China Plain (Daming [DM], Sheqi [SQ], Taihe [TH], and Tengzhou [TZ]). The relative abundances of the different groups are compared across **(A)** site, soil type (alkaline or acidic) and niche environment (rhizosphere or Bulk soil), **(B)** soil type, and **(C)** niche environment. The relative abundances are based on the frequencies of DNA sequences that could be classified to the class level. “Other” represents sequences that were unclassified and sequences that were present in amounts of less than 1% of the total.

After high-throughput sequencing, between 11,318 and 23,839 high-quality microeukaryotic sequences were obtained per sample. Of these, 99.9% were classified into a total of 1,034 distinct ASVs, including mostly fungi (95.3%), followed by other microbial eukaryotes such as Ciliophora (2.79%), Aphelidea (0.14%), and very few Incertae_Sedis, Amoebozoa, and Cercozoa (less than 0.01% in total). *Sordariomycetes*, *Dothideomycetes*, and *Tremellomycetes* dominated the assigned microeukaryotic classes (mainly fungi) and accounted for approximately 30.3, 13.6, and 5.82% of all ASVs sequences, respectively (Figure 1). The relative abundance of each microeukaryotic group varied among soil groups. For example, *Sordariomycetes* was highly abundant in alkaline and bulk soils (Figures 1B,C). Meanwhile, *Dothideomycetes* was less abundant in bulk soils than in rhizosphere soils (Figure 1C). *Tremellomycetes* was more abundant in acidic soils than in alkaline soils. The alpha diversity, which was represented by the observed species, was higher in alkaline soils than in acidic soils. Meanwhile, in bulk and rhizosphere soils, there was no significant difference in alpha diversity (Supplementary Figure 1).

### Soil Microeukaryotic Community Composition and Distribution Patterns

**FIGURE 2:**
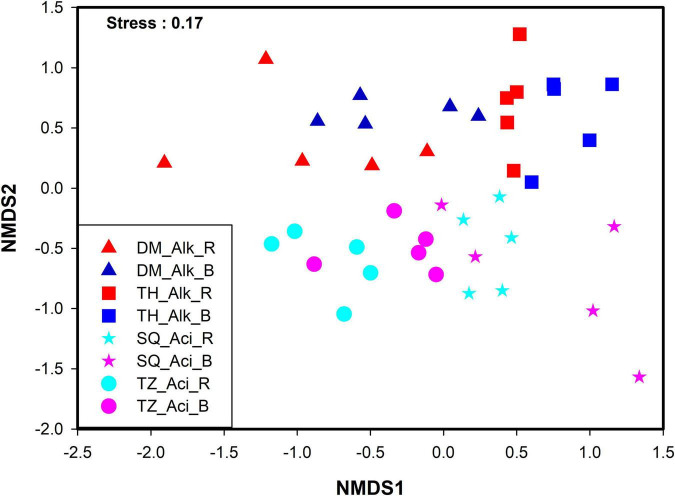
Non-metric multidimensional scaling (NMDS) ordinations showing the microeukaryotic community compositional dissimilarities among the four sampling sites. Triangles, Daming [DM]; squares, Taihe [TH]; stars, Sheqi [SQ]; circles, Tengzhou [TZ]; red, alkaline rhizosphere soil; blue, alkaline bulk soil; light blue, acidic rhizosphere soil; pink, acidic bulk soil.

The non-metric multidimensional scaling (NMDS) ordination plots of the soil microeukaryotic communities displayed clear patterns (Figure 2). The results showed that the microeukaryotic communities could significantly differ between alkaline and acidic soils (Adonis test, *F* = 4.17, R^2^ = 0.1, *P* < 0.001), and between bulk and rhizosphere soils (Adonis test, *F* = 1.70, R^2^ = 0.04, *P* < 0.001). In particular, the acidity and alkalinity of the soil had a stronger effect than the niche difference on the community composition (Figure 2).

To elucidate the relative roles of the environment and spatial distance on the microeukaryotic community, Mantel and partial Mantel tests were conducted. The results showed that both the soil pH (bulk: *r* = 0.49, *P* = 0.001; rhizosphere: *r* = 0.29, *P* = 0.001) and soil moisture (bulk: *r* = 0.28, *P* = 0.001; rhizosphere: *r* = 0.17, *P* = 0.026) significantly correlated with the microeukaryotic community composition in the bulk and rhizosphere soils. The soil pH had a stronger effect than the soil moisture (Table 1). The partial Mantel tests showed that soil pH and spatial distance had similar effects on the soil microeukaryotic community composition in the bulk soil. However, in the rhizosphere soil, spatial distance had a stronger effect than the soil pH.

**TABLE 1:** Mantel test results showing relationships between soil pH, soil moisture (SM), and eukaryotic community composition in rhizosphere and bulk soil.

Mantel test	Bulk soil		Rhizosphere	
	*r*	*p*	*r*	*p*
pH	0.49	0.001	0.29	0.001
SM	0.28	0.001	0.17	0.026
**Partial mantel**				
pH-distance	0.31	0.001	0.16	0.022
Distance-Ph	0.30	0.001	0.39	0.001

*Partial Mantel test showing the relative importance of the soil pH and spatial distance for the eukaryotic community composition.*

### Soil Microeukaryotic Assembly Processes

**FIGURE 3:**
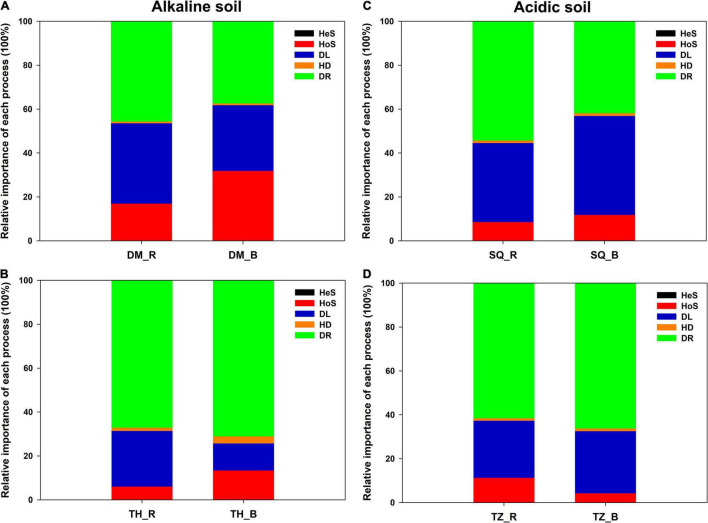
Relative importance of five ecological processes for the assembly of the microeukaryotic communities in the rhizosphere and bulk soils across the four sampling sites [**(A)**: Daming [DM], **(B)**: Taihe [TH], **(C)**: Sheqi [SQ], and **(D)**: Tengzhou [TZ]]. Ecological processes include: Dispersal limitation-DL, drift-DR, homogenous dispersal-HD, heterogeneous selection-HeS, and homogenous selection-HoS.

The iCAMP analysis revealed that DR was the most important among the five processes, with an average relative importance of 37.4–71.1% (Figure 3 and Supplementary Table 2). DL also provided a strong contribution to community assembly, with an average relative importance of 12.3–45.1% (Figure 3 and Supplementary Table 2). In particular, DL appeared to be slightly more important in the rhizosphere than in the bulk soil in alkaline soils (Figure 3). Meanwhile in acidic soils, the opposite trend was observed. Additionally, HoS was of slightly greater importance in bulk soils than in rhizosphere soils in DM, TH, and SQ, while it had less importance in TZ (Figure 3).

**FIGURE 4:**
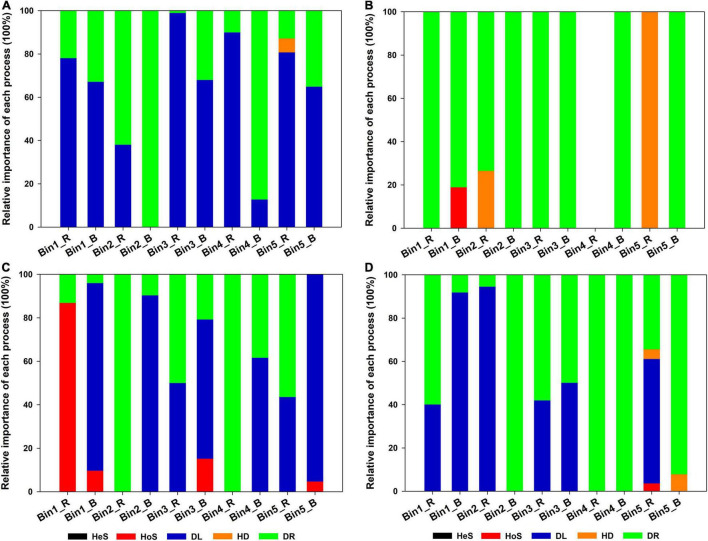
Relative importance of each ecological process for each iCAMP microeukaryotic lineage bin across the four sampling sites: **(A)** Sheqi (SQ), **(B)** Tengzhou (TZ), **(C)** Daming (DM), and **(D)** Taihe (TH). Only the five most abundant bins are shown. Source data can be found in Supplementary Table 2. For abbreviations, please see Figure 3.

The next step was to investigate the contribution of different assembly processes to individual lineages (i.e., bins). In this study, the observed 1,034 ASVs were divisible into 43 phylogenetic bins. The relative importance of a given assembly process was independent of the relative abundance in the bins (Figure 4). In addition, the contribution of each assembly process to a given bin varied according to the acidic or alkaline conditions of the soil and the niche environment. For example, for Bin1, DL provided a large contribution in the rhizosphere soil in SQ, but HoS was of greater importance in the rhizosphere soil in DM. For Bin2, DR contributed strongly in bulk soil in SQ, while DL showed greater importance in bulk soil in DM.

### Microeukaryotic Co-existence Networks

**FIGURE 5:**
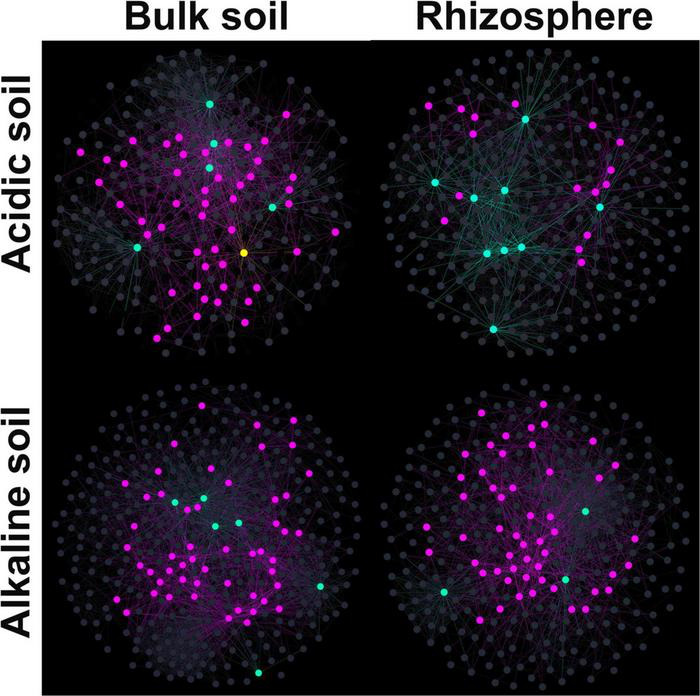
Co-existence networks for microeukaryotic communities in acidic bulk, acidic rhizosphere, alkaline bulk, and alkaline rhizosphere soil. The co-existence networks were constructed using the SparCC method to correlate amplified sequence variants (ASVs). For the node colors, yellow represents network hubs, light blue represents module hubs, purple represents connectors and dark gray represents peripherals.

Using SparCC correlation analysis, four association networks were constructed: acidic bulk soil, acidic rhizosphere soil, alkaline bulk soil, and alkaline rhizosphere soil (Figure 5). The topological features are shown in Table 2. Briefly, high proportions of positive links were found in the four ecological networks. Alkaline soil networks, especially the alkaline bulk soil network, showed greater complexity than acidic soil networks (Table 2).

**TABLE 2:** Topological features of the microeukaryotic co-existence networks in acidic bulk, acidic rhizosphere, alkaline bulk, and alkaline rhizosphere soils.

	Acidic bulk	Acidic rhizosphere	Alkaline bulk	Alkaline rhizosphere
Number of nodes	297	295	379	301
Number of edges	2,282	1,975	3,832	2,459
Transitivity (global)	0.40	0.36	0.40	0.44
Transitivity (average)	0.43	0.43	0.43	0.44
Centralization.degree	0.15	0.12	0.13	0.15
Centralization.betweeness	0.05	0.04	0.04	0.03
Centalization.evcent	0.84	0.84	0.82	0.84
Diameter	3.10	3.31	3.04	3.30
modularity	0.43	0.40	0.43	0.39
Complexity	7.68	6.69	10.11	8.17
Network hubs	0.00	0.00	0.00	0.00
Module hubs	0.02	0.03	0.02	0.01
Connectors	0.16	0.05	0.14	0.17
Peripherals	0.58	0.69	0.71	0.49
No function	0.24	0.22	0.14	0.33
Negative links	935	785	1,317	983
Positive links	1,347	1,190	2515	1,476
Negative links ratio	0.41	0.40	0.34	0.40
Positive links ratio	0.59	0.60	0.66	0.60

To identify the relative roles of the nodes in the networks, z and c scores were calculated for them for each soil type. Module hubs and connectors were frequently found in the four networks. However, a network hub was only found in the acidic bulk soil. Additionally, the acidic rhizosphere soil network harbored fewer connectors than the other three networks.

**FIGURE 6:**
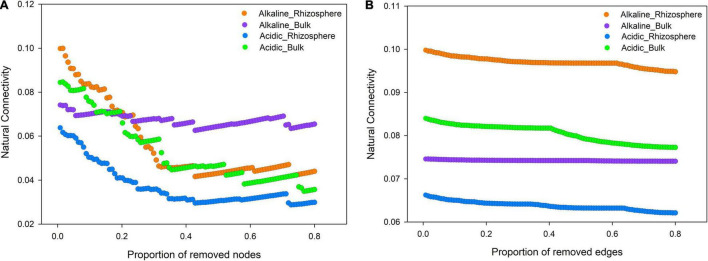
Robustness analysis of the rhizosphere and bulk soil microeukaryotic community networks in acidic and alkaline soils. Robustness is depicted as the relationship between natural connectivity and the proportion of excluded **(A)** nodes and **(B)** edges.

Natural connectivity analysis is a powerful method of investigating network robustness, which can, in turn, reflect network stability. Accordingly, the robustness of the four ecological networks was tested by altering the amplitude of natural connectivity via the deletion of nodes and edges (Figure 6). The results revealed that the rhizosphere community was more stable than the bulk soil community in alkaline soil. Meanwhile, the opposite trend was observed in acidic soil. This indicated that the adaptation mechanism of the microeukaryotes varied according to the soil pH conditions.

### The Role of Bins in the Network

To uncover the importance of the lineage bins in the networks, the bins were correlated to nodes in the network through ASVs ID. Finally, it was found that the five bins that were most influenced by each assembly process did not display important roles in the network (Table 3). For example, Bin1 was identified as peripheral in acidic bulk, acidic rhizosphere and alkaline rhizosphere soil, and only displayed connector function in alkaline bulk soil. However, the bins that were less influenced by the assembly processes than the top five bins, occupied important positions in the network such as module hubs and connectors. This suggested that the bins most tightly linked to the assembly processes may not play critical roles in the ecological network.

**TABLE 3:** The roles of different iCAMP microeukaryotic lineage bins within the microeukaryotic co-existence networks.

	ASVs ID	Category	Phylum	Class	Bin number
Acidic bulk	aaa048c42be0cab01d0a0d837f529f19	Connectors	Mucoromycota	Incertae_Sedis	Bin10
	747a57ff49b686a08dab407930cfc32a	Connectors	Ascomycota	Orbiliomycetes	Bin40
	e2eef71473c2fa43770949e5e98c751a	Connectors	Basidiomycota	Agaricomycetes	Bin24
	332dd2d96dfcb0fa96ad9d27e5ee6300	Connectors	Ascomycota	Eurotiomycetes	Bin37
	550c794a62fdc915b36b09cf87742cd2	Connectors	Mucoromycota	Incertae_Sedis	Bin8
	1fd56d5d031004ed0a24e326c6424439	Connectors	Chytridiomycota	Chytridiomycetes	Bin9
	b3b02416067dc2930e8d311a72df8df1	Connectors	Basidiomycota	Tremellomycetes	Bin25
	1f02799fb3678379114590dbab1b92d1	Connectors	Other	Other	Bin13
	c1a178d6fca2da1cc82b69f8879eaced	Connectors	Ascomycota	Sordariomycetes	Bin36
	7484ce605e5f93b6201a0aa0d8b50783	Module hubs	Ciliophora	Intramacronucleata	Bin16
	9a65e57e92f930920a83ad8dc210c46b	Module hubs	Ascomycota	Dothideomycetes	Bin34
	3960cab58a4fc018851850d9fa9768f0	Module hubs	Ascomycota	Sordariomycetes	Bin35
	a899e05cfae236d906be2ada77cca161	Module hubs	Ascomycota	Sordariomycetes	Bin33
	1711530852bf73eb7e54d4702da9990a	Network hubs	Ascomycota	Sordariomycetes	Bin31
	e0a3a17e1a2c4daac6b038b09014d841	Peripherals	Zoopagomycota	Incertae_Sedis	**Bin1**
Acidic rhizosphere	332dd2d96dfcb0fa96ad9d27e5ee6300	Connectors	Ascomycota	Eurotiomycetes	Bin37
	35b8439fe1ddb3d9b718efe25384b54b	Connectors	Ascomycota	Sordariomycetes	Bin28
	5641dead7620323c3a7ebdfa2f1a5dbe	Connectors	Ascomycota	Sordariomycetes	Bin32
	e2eef71473c2fa43770949e5e98c751a	Connectors	Basidiomycota	Agaricomycetes	Bin24
	0df00019868935ea2dfe1cf894dfda25	Module hubs	Ascomycota	Leotiomycetes	Bin43
	1711530852bf73eb7e54d4702da9990a	Module hubs	Ascomycota	Sordariomycetes	Bin31
	7484ce605e5f93b6201a0aa0d8b50783	Module hubs	Ciliophora	Intramacronucleata	Bin16
	9a65e57e92f930920a83ad8dc210c46b	Module hubs	Ascomycota	Dothideomycetes	Bin34
	7b1ce387ccb9a204c7e30fd70fe0d8e1	Peripherals	Mucoromycota	Incertae_Sedis	Bin2
	c48f844679ff23d1666a60afae02feff	Peripherals	Zoopagomycota	Incertae_Sedis	Bin3
	e0a3a17e1a2c4daac6b038b09014d841	Peripherals	Zoopagomycota	Incertae_Sedis	**Bin1**
Alkaline bulk	e0a3a17e1a2c4daac6b038b09014d841	Connectors	Zoopagomycota	Incertae_Sedis	**Bin1**
	b3b02416067dc2930e8d311a72df8df1	Connectors	Basidiomycota	Tremellomycetes	Bin25
	1f02799fb3678379114590dbab1b92d1	Connectors	Other	Other	Bin13
	1711530852bf73eb7e54d4702da9990a	Module hubs	Ascomycota	Sordariomycetes	Bin31
	c1a178d6fca2da1cc82b69f8879eaced	Module hubs	Ascomycota	Sordariomycetes	Bin36
	7b1ce387ccb9a204c7e30fd70fe0d8e1	Peripherals	Mucoromycota	Incertae_Sedis	**Bin2**
	3dc85ce8c589f8e7a271e13f58b0ad79	Peripherals	Schizoplasmodiida	Schizoplasmodiida	**Bin4**
Alkaline rhizosphere	747a57ff49b686a08dab407930cfc32a	Connectors	Ascomycota	Orbiliomycetes	Bin40
	19c83a40d6649ec2c9c5344f32675165	Connectors	Ascomycota	Pezizomycetes	Bin42
	332dd2d96dfcb0fa96ad9d27e5ee6300	Connectors	Ascomycota	Eurotiomycetes	Bin37
	7f378e662b7de4a5bcac7b4d0db0096e	Connectors	Ascomycota	Eurotiomycetes	Bin39
	35b8439fe1ddb3d9b718efe25384b54b	Connectors	Ascomycota	Sordariomycetes	Bin28
	550c794a62fdc915b36b09cf87742cd2	Connectors	Mucoromycota	Incertae_Sedis	Bin8
	e5dd42d5151462e8a9a4f68c2bd63286	Connectors	Basidiomycota	Agaricomycetes	Bin22
	5641dead7620323c3a7ebdfa2f1a5dbe	Connectors	Ascomycota	Sordariomycetes	Bin32
	a899e05cfae236d906be2ada77cca161	Connectors	Ascomycota	Sordariomycetes	Bin33
	ad4b6e5052089eaf0049964c93627677	Connectors	Ascomycota	Dothideomycetes	Bin29
	1f02799fb3678379114590dbab1b92d1	Connectors	Other	Other	Bin13
	1711530852bf73eb7e54d4702da9990a	Module hubs	Ascomycota	Sordariomycetes	Bin31
	7b1ce387ccb9a204c7e30fd70fe0d8e1	Peripherals	Mucoromycota	Incertae_Sedis	Bin2
	e0a3a17e1a2c4daac6b038b09014d841	Peripherals	Zoopagomycota	Incertae_Sedis	**Bin1**

*Bold values mean that they make a greater contribution.*

## Discussion

The spatial distribution, assembly processes, and co-existence networks of microeukaryotic community were revealed in the earing and flowering stage of wheat (late April), which is crucial for microbes (Roesti et al., 2006). Generally, fungi was the dominant microeukaryotic group in the soil, and it was also evident in many studies (Anderson et al., 2003; Tedersoo et al., 2014; Shi et al., 2019a). Our findings revealed that the pH of the soil displayed a stronger effect on the soil microeukaryotic community composition than niche differences (bulk vs. rhizosphere). This indicated the powerful regulation function of soil pH in nutrients storing and supplying (Slessarev et al., 2016), which in turn presented a strong effect on microbes. Therefore, the analyses of assembly processes and co-existence networks were firstly grouped by acidity and alkalinity, and then divided by bulk and rhizosphere. Generally, DR was the dominant process driving microeukaryotic community assembly across all soils. Additionally, the contribution of the different assembly processes in each bin varied according to the soil pH and niche environment, reflecting the importance of microbial species corresponding to environment or soil pH conditions. Alkaline soil networks showed greater complexity than acidic soil networks, and the rhizosphere community was more stable than the bulk soil community in alkaline soil. Consistent with our first hypothesis, the soil pH played an important role in driving microeukaryotic community distribution. Using a global set of samples, Aslani et al. (2021) also found that soil pH was a main driver determining soil microeukaryotic community structure. Numerous studies have reported the primary role of soil pH in shaping soil bacterial and fungal distribution patterns (Fierer, 2017). For example, Shen et al. (2013) found that soil pH could drive the spatial distribution of soil bacterial communities with respect to elevation on Changbai Mountain. Lauber et al. (2009) reported that soil pH was a predictor of soil bacterial community structure at the continental scale. More recently, Shi et al. (2018, 2021) revealed that soil bacterial and fungal community structures could both be determined by soil pH in the North China Plain. Using a natural system, significant correlations were found between protist ß diversity and phosphorous (Logares et al., 2018). While, across a broad geographic range, soil protists were found marginally influenced by pH (Bates et al., 2013). Given that soil pH is critical in soil nutrient cycling (Fierer, 2017), both the prokaryotes and eukaryotes are involved in the cycle all the time, therefore the important role of pH on the soil microbial eukaryotes is obvious.

Our results confirmed that drift played a dominant role in controlling microeukaryotic community assembly. These results are consistent with the global-scale investigation of soil microeukaryotes (Aslani et al., 2021). Drift reflects the influence of random demographic variability, including birth, death and migration rates, on the microbial community (Martiny et al., 2006). Drift results in a high dispersal rate, which can homogenize the community and thus form weaker distance—decay patterns (Vellend, 2010). Some studies found drift process was important for microeukaryotes (Orrock and Watling, 2010; Powell et al., 2015; Logares et al., 2018; Fodelianakis et al., 2021), but not for prokaryotes. Here, we speculated that compared to bacteria, fungi or protist species have larger body size, which is positively correlated with the proportion of the drift (Aslani et al., 2021). On the other hand, larger body size species which present higher birth or death rate will be shown as higher proportion of drift in ecological process (Logares et al., 2018). Different from prokaryotes such as bacteria with lighter mass and smaller body size (Smith et al., 2013), for the fungi and protists, dispersal limitation presents stronger effect on their assembly process due to their low mobility. In our study, contrasting microeukaryotic community assembly patterns were found in alkaline soil and acidic soils. Possibly, the reason is that the filtering effect of plant roots in alkaline soils is stronger than that in acidic soils (Fan et al., 2017; Nuccio et al., 2020). This suggested that the niche environment and soil pH condition jointly affected soil microeukaryotic community assembly in the agricultural ecosystem, indicating the importance of habitats and environments in mediating assembly process of soil microbes.

The results of the present study showed that the microeukaryotic networks of the acidic soils were less complex than those in the alkaline soils, indicating the importance of soil pH in determining the microbial association network. Furthermore, the stability, which was represented by the robustness, was greater in alkaline soil than in acidic soils. These findings suggest that the capacity of a soil microeukaryotic community to maintain stability was not independent of its complexity, but also related to the soil pH conditions. Under permafrost conditions, Wu et al. (2021) also found that microbial (including bacteria and fungi) network complexity was associated with community stability. However, they found an opposing trend; the greater the complexity, the lower the stability. An explanation for these conflicting results could be that the larger body size of the microeukaryotes means that they respond differently from soil bacteria and fungi to their environments (Aslani et al., 2021). Indeed, wheat rhizosphere soil has previously been found to harbor a less complex community and more stable microbial association network in North China Plain soils (Fan et al., 2018). This could be due to the stronger filtering effect of rhizosphere which has stronger ability in recruiting beneficial microbes, and thus builds more stable environment (Thebault and Fontaine, 2010; de Vries et al., 2020). In this study, although we found high abundance of fungal sequences in the soil and few other microbial eukaryotic species, all microbial eukaryotic sequences were analysed for diversity, community characteristics and network associations. Compare to other microeukaryotes, higher abundance fungi contributes a great deal to the ecological functions (e.g., litter decomposition, carbon and nitrogen cycling) (Tedersoo et al., 2014). Our findings provide the first insight into microeukaryotic community (mainly fungi) complexity and stability, and the relationship between them, in agricultural ecosystems.

## Concluding Remarks

Our results indicated the critical role of soil pH in determining community distribution patterns, assembly processes and co-existence networks of soil microeukaryotes in wheat fields of the North China Plain. Furthermore, we identified the dominant role of drift in controlling microeukaryotic community assembly. Finally, we found greater microeukaryotic community complexity was associated with a more stable community in crop rhizosphere. These findings broaden our understanding about the important influence of soil pH in soil microbes from procaryotic to eukaryotic microbes, which has implications for microbial functioning under various agricultural practices.

## Data Availability Statement

The datasets presented in this study can be found in online repositories. The names of the repository/repositories and accession number(s) can be found below: www.ncbi.nlm.nih.gov/sra/, SRP347607.

## Author Contributions

HC designed the study. YS, MX, YZ, and LC performed the research and analyzed the data. YS and HC wrote, edited, and finalized the manuscript. All authors contributed to the article and approved the submitted version.

## Conflict of Interest

The authors declare that the research was conducted in the absence of any commercial or financial relationships that could be construed as a potential conflict of interest.

## Publisher’s Note

All claims expressed in this article are solely those of the authors and do not necessarily represent those of their affiliated organizations, or those of the publisher, the editors and the reviewers. Any product that may be evaluated in this article, or claim that may be made by its manufacturer, is not guaranteed or endorsed by the publisher.

## Footnotes

1
https://unite.ut.ee/

2
https://igraph.org/r/

3
http://gephi.github.io/

## References

[B1] Albert R. Jeong H. Barabasi A. L. (2000). Error and attack tolerance of complex networks. *Nature* 406 378–382. 10.1038/35019019 10935628

[B2] Amir A. McDonald D. Navas-Molina J. A. Kopylova E. Morton J. T. Zech Xu Z. (2017). Deblur rapidly resolves single-nucleotide community sequence patterns. *mSystems* 2 e00191–16. 10.1128/mSystems.00191-16 28289731 PMC5340863

[B3] Anderson I. C. Campbell C. D. Prosser J. I. (2003). Potential bias of fungal 18S rDNA and internal transcribed spacer polymerase chain reaction primers for estimating fungal biodiversity in soil. *Environ. Microbiol.* 5 36–47. 10.1046/j.1462-2920.2003.00383.x 12542711

[B4] Aslani F. Geisen S. Ning D. Tedersoo L. Bahram M. de Vries F. (2021). Towards revealing the global diversity and community assembly of soil eukaryotes. *Ecol. Lett.* 25 65–76. 10.1111/ele.13904 34697894

[B5] Bates S. T. Clemente J. C. Flores G. E. Walters W. A. Parfrey L. W. Knight R. (2013). Global biogeography of highly diverse protistan communities in soil. *ISME J.* 7 652–659. 10.1038/ismej.2012.147 23235291 PMC3578557

[B6] Bolyen E. Rideout J. R. Dillon M. R. (2019). Reproducible, interactive, scalable and extensible microbiome data science using QIIME 2. *Nat. Biotechnol.* 37 1091–1091. 10.1038/s41587-019-0209-9 31341288 PMC7015180

[B7] Cardinale M. Grube M. Erlacher A. Quehenberger J. Berg G. (2015). Bacterial networks and co-occurrence relationships in the lettuce root microbiota. *Environ. Microbiol* 17 239–252. 10.1111/1462-2920.12686 25367329

[B8] Coux C. Rader R. Bartomeus I. Tylianakis J. M. (2016). Linking species functional roles to their network roles. *Ecol. Lett.* 19 762–770. 10.1111/ele.12612 27169359

[B9] Crowther T. W. van den Hoogen J. Wan J. Mayes M. A. Keiser A. D. Mo L. (2019). The global soil community and its influence on biogeochemistry. *Science* 365:772. 10.1126/science.aav0550 31439761

[B10] de Vries F. T. Griffiths R. I. Knight C. G. Nicolitch O. Williams A. (2020). Harnessing rhizosphere microbiomes for drought-resilient crop production. *Science* 368 270–274. 10.1126/science.aaz5192 32299947

[B11] Delgado-Baquerizo M. Oliverio A. M. Brewer T. E. Benavent-Gonzalez A. Eldridge D. J. Bardgett R. D. (2018). A global atlas of the dominant bacteria found in soil. *Science* 359:320. 10.1126/science.aap9516 29348236

[B12] Delgado-Baquerizo M. Reich P. B. Trivedi C. Eldridge D. J. Abades S. Alfaro F. D. (2020). Multiple elements of soil biodiversity drive ecosystem functions across biomes. *Nat. Ecol. Evol.* 4 210–220. 10.1038/s41559-019-1084-y 32015427

[B13] Dini-Andreote F. Stegen J. C. van Elsas J. D. Salles J. F. (2015). Disentangling mechanisms that mediate the balance between stochastic and deterministic processes in microbial succession. *Proc. Natl. Acad. Sci. U.S.A.* 112 E1326–E1332. 10.1073/pnas.1414261112 25733885 PMC4371938

[B14] Donn S. Kirkegaard J. A. Perera G. Richardson A. E. Watt M. (2015). Evolution of bacterial communities in the wheat crop rhizosphere. *Environ. Microbiol* 17 610–621. 10.1111/1462-2920.12452 24628845

[B15] Fan K. K. Cardona C. Li Y. T. Shi Y. Xiang X. J. Shen C. C. (2017). Rhizosphere-associated bacterial network structure and spatial distribution differ significantly from bulk soil in wheat crop fields. *Soil Biol. Biochem.* 113 275–284. 10.1016/j.soilbio.2017.06.020

[B16] Fan K. K. Weisenhorn P. Gilbert J. A. Chu H. Y. (2018). Wheat rhizosphere harbors a less complex and more stable microbial co-occurrence pattern than bulk soil. *Soil Biol. Biochem.* 125 251–260. 10.1016/j.soilbio.2018.07.022

[B17] Faust K. Raes J. (2012). Microbial interactions: from networks to models. *Nat. Rev. Microbiol.* 10 538–550. 10.1038/nrmicro2832 22796884

[B18] Faust K. Lima-Mendez G. Lerat J. S. Sathirapongsasuti J. F. Knight R. Huttenhower C. (2015). Cross-biome comparison of microbial association networks. *Front. Microbiol.* 6:1200. 10.3389/fmicb.2015.01200 26579106 PMC4621437

[B19] Feng M. M. Adams J. M. Fan K. K. Shi Y. Sun R. B. Wang D. Z. (2018). Long-term fertilization influences community assembly processes of soil diazotrophs. *Soil Biol. Biochem.* 126 151–158. 10.1016/j.soilbio.2018.08.021

[B20] Feng Y. Chen R. Stegen J. C. Guo Z. Zhang J. Li Z. (2018). Two key features influencing community assembly processes at regional scale: initial state and degree of change in environmental conditions. *Mol. Ecol. Resour.* 27 5238–5251. 10.1111/mec.14914 30368967

[B21] Fierer N. (2017). Embracing the unknown: disentangling the complexities of the soil microbiome. *Nat. Rev. Microbiol.* 15 579–590. 10.1038/nrmicro.2017.87 28824177

[B22] Fierer N. Jackson R. B. (2006). The diversity and biogeography of soil bacterial communities. *Proc. Natl. Acad. Sci. U.S.A.* 103 626–631. 10.1073/pnas.0507535103 16407148 PMC1334650

[B23] Fodelianakis S. Valenzuela-Cuevas A. Barozzi A. Daffonchio D. (2021). Direct quantification of ecological drift at the population level in synthetic bacterial communities. *ISME J.* 15 55–66. 10.1038/s41396-020-00754-4 32855435 PMC7852547

[B24] Giner C. R. Balague V. Krabberod A. K. Ferrera I. Rene A. Garces E. (2018). Quantifying long-term recurrence in planktonic microbial eukaryotes. *Mol. Ecol.* 28 923–935. 10.1111/mec.14929 30411822

[B25] Jeong S. J. Ho C. H. Piao S. L. Kim J. Ciais P. Lee Y. B. (2014). Effects of double cropping on summer climate of the North China Plain and neighbouring regions. *Nat. Clim. Chang.* 4 615–619. 10.1038/NCLIMATE2266

[B26] Jiao S. Lu Y. H. (2020). Soil pH and temperature regulate assembly processes of abundant and rare bacterial communities in agricultural ecosystems. *Environ. Microbiol.* 22 1052–1065. 10.1111/1462-2920.14815 31599105

[B27] Jordan F. (2009). Keystone species and food webs. *Philos Trans. R. Soc. B Biol. Sci.* 364 1733–1741. 10.1098/rstb.2008.0335 19451124 PMC2685432

[B28] Lauber C. L. Hamady M. Knight R. Fierer N. (2009). Pyrosequencing-based assessment of soil pH as a predictor of soil bacterial community structure at the continental scale. *Appl. Environ. Microbiol.* 75 5111–5120. 10.1128/AEM.00335-09 19502440 PMC2725504

[B29] Li E. de Jonge R. Liu C. Jiang H. Friman V.-P. Pieterse C. M. J. (2021). Rapid evolution of bacterial mutualism in the plant rhizosphere. *Nat. Commun* 12:3829. 10.1038/s41467-021-24005-y 34158504 PMC8219802

[B30] Lima-Mendez G. Faust K. Henry N. Decelle J. Colin S. Carcillo F. (2015). Determinants of community structure in the global plankton interactome. *Science* 348:6237. 10.1126/science.1262073 25999517

[B31] Liu Y. R. Eldridge D. J. Zeng X. M. Wang J. Singh B. K. Delgado-Baquerizo M. (2021). Global diversity and ecological drivers of lichenised soil fungi. *New Phytol.* 231 1210–1219. 10.1111/nph.17433 33914920

[B32] Logares R. Tesson S. V. Canback B. Pontarp M. Hedlund K. Rengefors K. (2018). Contrasting prevalence of selection and drift in the community structuring of bacteria and microbial eukaryotes. *Environ. Microbiol.* 20 2231–2240. 10.1111/1462-2920.14265 29727053

[B33] Ma B. Wang Y. L. Ye S. D. Liu S. Stirling E. Gilbert J. A. (2020). Earth microbial co-occurrence network reveals interconnection pattern across microbiomes. *Microbiome* 8:82. 10.1186/s40168-020-00857-2 32498714 PMC7273686

[B34] Ma B. Wang H. Dsouza M. Lou J. He Y. Dai Z. (2016). Geographic patterns of co-occurrence network topological features for soil microbiota at continental scale in eastern China. *ISME J*. 10 1891–1901. 10.1038/ismej.2015.261 26771927 PMC5029158

[B35] Martiny J. B. H. Bohannan B. J. M. Brown J. H. Colwell R. K. Fuhrman J. A. Green J. L. (2006). Microbial biogeography: putting microorganisms on the map. *Nat. Rev. Microbiol.* 4 102–112. 10.1038/nrmicro1341 16415926

[B36] May M.R. (1973). *Stability and Complexity in Model Ecosystems.* Princeton, NJ: Princeton University Press.

[B37] Neutel A. M. Heesterbeek J. A. P. de Ruiter P. C. (2002). Stability in real food webs: weak links in long loops. *Science* 296 1120–1123. 10.1126/science.1068326 12004131

[B38] Ning D. Yuan M. Wu L. Zhang Y. Guo X. Zhou X. (2020). A quantitative framework reveals ecological drivers of grassland microbial community assembly in response to warming. *Nat. Commun.* 11:4717. 10.1038/s41467-020-18560-z 32948774 PMC7501310

[B39] Nuccio E. E. Starr E. Karaoz U. Brodie E. L. Zhou J. Tringe S. (2020). Niche differentiation is spatially and temporally regulated 1 in the rhizosphere. *ISME J.* 14 999–1014. 10.1038/s41396-019-0582-x 31953507 PMC7082339

[B40] Oliverio A. M. Geisen S. Delgado-Baquerizo M. Maestre F. T. Turner B. L. Fierer N. (2020). The global-scale distributions of soil protists and their contributions to belowground systems. *Sci. Adv.* 6:eaax8787. 10.1126/sciadv.aax8787 32042898 PMC6981079

[B41] Orrock J. L. Watling J. I. (2010). Local community size mediates ecological drift and competition in metacommunities. *Proc. Biol. Sci* 277 2185–2191. 10.1098/rspb.2009.234 20236983 PMC2880146

[B42] Peng G. S. Wu J. (2016). Optimal network topology for structural robustness based on natural connectivity. *Phys. A Stat. Mech. Appl.* 443 212–220. 10.1016/j.physa.2015.09.023

[B43] Piao S. Ciais P. Huang Y. Shen Z. Peng S. Li J. (2010). The impacts of climate change on water resources and agriculture in China. *Nature* 467 43–51. 10.1038/nature09364 20811450

[B44] Pineda A. Kaplan I. Bezemer T. M. (2017). Steering soil microbiomes to suppress aboveground insect pests. *Trends Plant. Sci.* 22 770–778. 10.1016/j.tplants.2017.07.002 28757147

[B45] Powell J. R. Karunaratne S. Campbell C. D. Yao H. Robinson L. Singh B. K. (2015). Deterministic processes vary during community assembly for ecologically dissimilar taxa. *Nat. Commun* 6:8444. 10.1038/ncomms9444 26436640 PMC4600744

[B46] Roesti D. Gaur R. Johri B. N. Imfeld G. Sharma S. Kawaljeet K. (2006). Plant growth stage, fertiliser management and bio-inoculation of arbuscular mycorrhizal fungi and plant growth promoting rhizobacteria affect the rhizobacterial community structure in rain-fed wheat fields. *Soil Biol. Biochem.* 38 1111–1120. 10.1016/j.soilbio.2005.09.010

[B47] Rousk J. Baath E. Brookes P. C. Lauber C. L. Lozupone C. Caporaso J. G. (2010). Soil bacterial and fungal communities across a pH gradient in an arable soil. *ISME J*. 4 1340–1351. 10.1038/ismej.2010.58 20445636

[B48] Shen C. C. Xiong J. B. Zhang H. Y. Feng Y. Z. Lin X. G. Li X. Y. (2013). Soil pH drives the spatial distribution of bacterial communities along elevation on Changbai Mountain. *Soil Biol. Biochem.* 57 204–211. 10.1016/j.soilbio.2012.07.013

[B49] Shi Y. Dang K. K. Dong Y. H. Feng M. M. Wang B. R. Li J. G. (2019a). Soil fungal community assembly processes under long-term fertilization. *Eur. J. Soil. Sci* 71 716–726. 10.1111/ejss.12902

[B51] Shi Y. Fan K. Li Y. Yang T. He J.-S. Chu H. (2019b). Archaea enhance the robustness of microbial co-occurrence networks in tibetan plateau soils. *Soil. Sci. Soc. Am. J.* 83 1093–1099. 10.2136/sssaj2018.11.0426

[B50] Shi Y. Delgado-Baquerizo M. Li Y. Yang Y. Zhu Y.-G. Peñuelas J. (2020). Abundance of kinless hubs within soil microbial networks are associated with high functional potential in agricultural ecosystems. *Environ. Int.* 142:105869. 10.1016/j.envint.2020.105869 32593837

[B52] Shi Y. Li Y. T. Xiang X. J. Sun R. B. Yang T. He D. (2018). Spatial scale affects the relative role of stochasticity versus determinism in soil bacterial communities in wheat fields across the North China Plain. *Microbiome* 6:27. 10.1186/s40168-018-0409-4 29402331 PMC5799910

[B53] Shi Y. Li Y. Yang T. Chu H. (2021). Threshold effects of soil pH on microbial co-occurrence structure in acidic and alkaline arable lands. *Sci. Total Environ.* 800:149592. 10.1016/j.scitotenv.2021.149592 34426307

[B54] Slessarev E. W. Lin Y. Bingham N. L. Johnson J. E. Dai Y. Schimel J. P. (2016). Water balance creates a threshold in soil pH at the global scale. *Nature* 540 567–569. 10.1038/nature20139 27871089

[B55] Smith D. J. Timonen H. J. Jaffe D. A. Griffin D. W. Birmele M. N. Perry K. D. (2013). Intercontinental dispersal of bacteria and archaea by transpacific winds. *Appl. Environ. Microbiol.* 79 1134–1139. 10.1128/AEM.03029-12 23220959 PMC3568602

[B56] Soudzilovskaia N. A. van Bodegom P. M. Terrer C. Zelfde M. V. McCallum I. Luke McCormack M. (2019). Global mycorrhizal plant distribution linked to terrestrial carbon stocks. *Nat. Commun.* 10:5077. 10.1038/s41467-019-13019-2 31700000 PMC6838125

[B57] Stegen J. C. Freestone A. L. Crist T. O. Anderson M. J. Chase J. M. Comita L. S. (2013). Stochastic and deterministic drivers of spatial and temporal turnover in breeding bird communities. *Glob. Ecol. Biogeogr.* 22 202–212. 10.1111/j.1466-8238.2012.00780.x

[B58] Tedersoo L. Bahram M. Polme S. Koljalg U. Yorou N. S. Wijesundera R. (2014). Global diversity and geography of soil fungi. *Science* 346:1078. 10.1126/science.1256688 25430773

[B59] Thebault E. Fontaine C. (2010). Stability of ecological communities and the architecture of mutualistic and trophic networks. *Science* 329 853–856. 10.1126/science.1188321 20705861

[B60] Vellend M. (2010). Conceptual synthesis in community ecology. *Q. Rev. Biol.* 85 183–206. 10.1086/652373 20565040

[B61] Weiss S. Van Treuren W. Lozupone C. Faust K. Friedman J. Deng Y. (2016). Correlation detection strategies in microbial data sets vary widely in sensitivity and precision. *ISME J.* 10 1669–1681. 10.1038/ismej.2015.235 26905627 PMC4918442

[B62] Whitaker R. J. Grogan D. W. Taylor J. W. (2003). Geographic barriers isolate endemic populations of hyperthermophilic archaea. *Science* 301 976–978. 10.1126/science.1086909 12881573

[B63] Wu M. H. Chen S. Y. Chen J. W. Xue K. Chen S. L. Wang X. M. (2021). Reduced microbial stability in the active layer is associated with carbon loss under alpine permafrost degradation. *Proc. Natl. Acad. Sci. U.S.A.* 118:e2025321118. 10.1073/pnas.2025321118 34131077 PMC8237688

[B64] Xiong W. Li R. Guo S. Karlsson I. Jiao Z. X. Xun W. B. (2019). Microbial amendments alter protist communities within the soil microbiome. *Soil Biol. Biochem.* 135 379–382. 10.1016/j.soilbio.2019.05.025

[B65] Xiong W. Song Y. Yang K. Gu Y. Wei Z. Kowalchuk G. A. (2020). Rhizosphere protists are key determinants of plant health. *Microbiome* 8:27. 10.1186/s40168-020-00799-9 32127034 PMC7055055

[B66] Yang T. Tedersoo L. Soltis P. S. Soltis D. E. Gilbert J. A. Sun M. (2019). Phylogenetic imprint of woody plants on the soil mycobiome in natural mountain forests of eastern China. *ISME J.* 13 686–697. 10.1038/s41396-018-0303-x 30353037 PMC6461945

[B67] Zhang B. Zhang J. Liu Y. Shi P. Wei G. (2018). Co-occurrence patterns of soybean rhizosphere microbiome at a continental scale. *Soil Biol. Biochem.* 118 178–186. 10.1016/j.soilbio.2017.12.011

[B68] Zhang L. Zhou J. George T. S. Limpens E. Feng G. (2022). Arbuscular mycorrhizal fungi conducting the hyphosphere bacterial orchestra. *Trends Plant. Sci.* 27 402–411. 10.1016/j.tplants.2021.10.008 34782247

[B69] Zhang W. J. Pan Y. B. Yang J. Chen H. H. Holohan B. Vaudrey J. (2018). The diversity and biogeography of abundant and rare intertidal marine microeukaryotes explained by environment and dispersal limitation. *Environ. Microbiol* 20 462–476. 10.1111/1462-2920.13916 28881067

[B70] Zhou J. Z. Deng Y. Zhang P. Xue K. Liang Y. T. Van Nostrand J. D. (2014). Stochasticity, succession, and environmental perturbations in a fluidic ecosystem. *Proc. Natl. Acad. Sci.U.S.A.* 111 E836–E845. 10.1073/pnas.1324044111 24550501 PMC3948316

[B71] Zhou J. Kang S. Schadt C. W. Garten C. T. (2008). Spatial scaling of functional gene diversity across various microbial taxa. *Proc. Natl. Acad. Sci. U.S.A.* 105 7768–7773. 10.1073/pnas.0709016105 18509054 PMC2409408

[B72] Zhu A. Zhang J. Zhao B. Cheng Z. Li L. (2005). Water balance and nitrate leaching losses under intensive crop production with Ochric Aquic Cambosols in North China Plain. *Environ. Int.* 31 904–912. 10.1016/j.envint.2005.05.038 15990170

