# Supplementary material for: Soil pH Determines the Spatial Distribution, Assembly Processes, and Co-existence Networks of Microeukaryotic Community in Wheat Fields of the North China Plain
Source: Front Microbiol. 2022 Jul 25;13:911116. doi: 10.3389/fmicb.2022.911116 (PMC9358722; doi:10.3389/fmicb.2022.911116)
Supplement: Supplementary file 1 [file Data_Sheet_1.docx]

Figure S1 Sampling site (A) and Conceptual sampling plot (B) in each site.


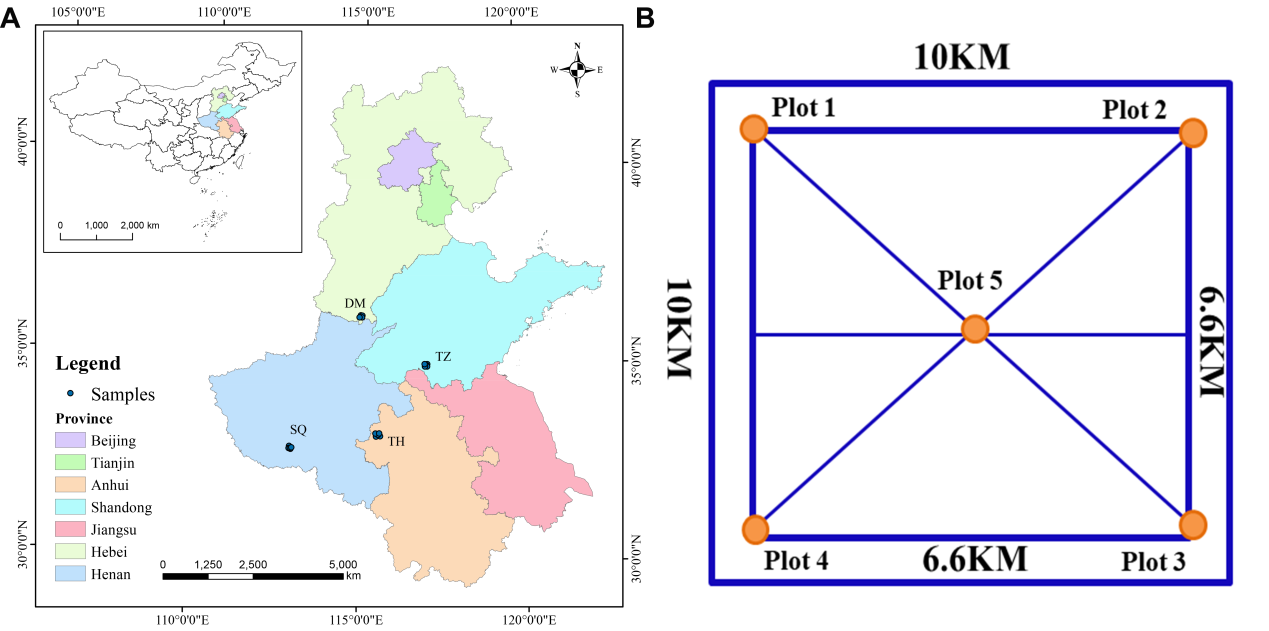


Figure S2 Observed species of the eukaryotic soil microbes grouped by acidic and alkaline, bulk and rhizosphere.


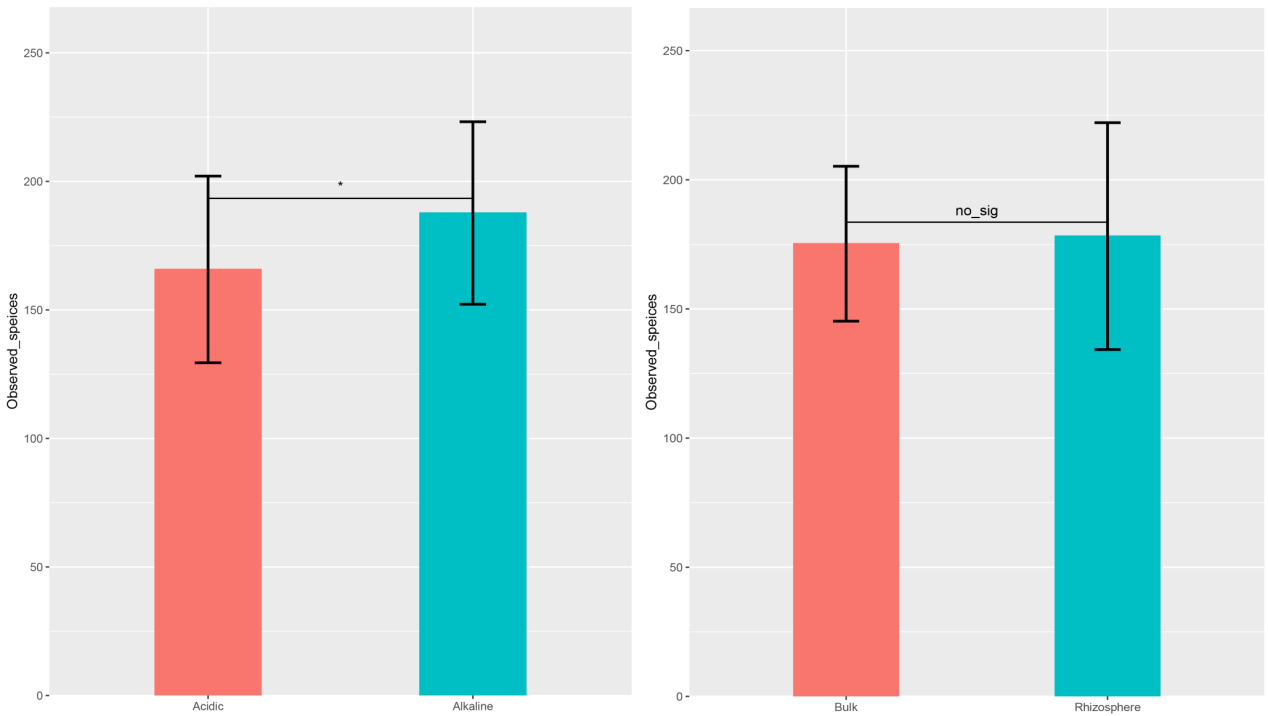


Table S1 Location information and soil variables. (SM: soil moisture)

|  | Long | Lat | pH | SM |
| --- | --- | --- | --- | --- |
| DM-rhizosphere1 | 114.99 | 36.24 | 7.56 | 0.01 |
| DM-rhizosphere2 | 114.99 | 36.29 | 7.53 | 0.09 |
| DM-rhizosphere3 | 115.03 | 36.28 | 7.48 | 0.10 |
| DM-rhizosphere4 | 115.02 | 36.24 | 7.94 | 0.11 |
| DM-rhizosphere5 | 114.94 | 36.24 | 8.19 | 0.10 |
| DM-non rhizosphere1 | 114.99 | 36.24 | 7.88 | 0.16 |
| DM-non rhizosphere2 | 114.99 | 36.29 | 7.95 | 0.11 |
| DM-non rhizosphere3 | 115.03 | 36.28 | 8.03 | 0.09 |
| DM-non rhizosphere4 | 115.02 | 36.24 | 7.88 | 0.12 |
| DM-non rhizosphere5 | 114.94 | 36.24 | 8.20 | 0.10 |
| SQ-rhizosphere1 | 112.90 | 32.93 | 6.70 | 0.23 |
| SQ-rhizosphere2 | 112.89 | 32.87 | 6.63 | 0.24 |
| SQ-rhizosphere3 | 112.95 | 32.85 | 6.16 | 0.22 |
| SQ-rhizosphere4 | 112.93 | 32.88 | 6.02 | 0.22 |
| SQ-rhizosphere5 | 112.97 | 32.89 | 5.42 | 0.20 |
| SQ-non rhizosphere1 | 112.90 | 32.93 | 5.11 | 0.18 |
| SQ-non rhizosphere2 | 112.89 | 32.87 | 5.80 | 0.20 |
| SQ-non rhizosphere3 | 112.95 | 32.85 | 5.92 | 0.21 |
| SQ-non rhizosphere4 | 112.93 | 32.88 | 5.61 | 0.20 |
| SQ-non rhizosphere5 | 112.97 | 32.89 | 4.69 | 0.20 |
| TH-rhizosphere1 | 115.51 | 33.22 | 8.08 | 0.12 |
| TH-rhizosphere2 | 115.64 | 33.23 | 8.06 | 0.13 |
| TH-rhizosphere3 | 115.58 | 33.26 | 8.01 | 0.12 |
| TH-rhizosphere4 | 115.50 | 33.30 | 8.06 | 0.14 |
| TH-rhizosphere5 | 115.61 | 33.30 | 8.05 | 0.14 |
| TH-non rhizosphere1 | 115.51 | 33.22 | 7.51 | 0.14 |
| TH-non rhizosphere2 | 115.64 | 33.23 | 7.83 | 0.15 |
| TH-non rhizosphere3 | 115.58 | 33.26 | 7.87 | 0.14 |
| TH-non rhizosphere4 | 115.50 | 33.30 | 7.99 | 0.16 |
| TH-non rhizosphere5 | 115.61 | 33.30 | 7.80 | 0.11 |
| TZ-rhizosphere1 | 117.06 | 35.07 | 5.32 | 0.07 |
| TZ-rhizosphere2 | 117.04 | 35.04 | 5.90 | 0.06 |
| TZ-rhizosphere3 | 117.06 | 35.01 | 5.28 | 0.06 |
| TZ-rhizosphere4 | 116.99 | 35.01 | 5.44 | 0.07 |
| TZ-rhizosphere5 | 116.98 | 35.07 | 4.72 | 0.05 |
| TZ-non rhizosphere1 | 117.06 | 35.07 | 5.54 | 0.08 |
| TZ-non rhizosphere2 | 117.04 | 35.04 | 5.69 | 0.09 |
| TZ-non rhizosphere3 | 117.06 | 35.01 | 5.68 | 0.06 |
| TZ-non rhizosphere4 | 116.99 | 35.01 | 5.54 | 0.11 |
| TZ-non rhizosphere5 | 116.98 | 35.07 | 5.09 | 0.04 |

Table S2 Relative importance of five ecological processes on the eukaryotic microbial commmunity in rhizosphere and bulk soils across the four sites. _B means bulk soil. _R means rhizosphere soil.

| Group | HeS | HoS | DL | HD | DR |
| --- | --- | --- | --- | --- | --- |
| TZ_B | 0.08 | 4.21 | 28.24 | 1.24 | 66.24 |
| SQ_B | 0.19 | 11.64 | 45.06 | 1.31 | 41.81 |
| **Acidic_B** | 0.13 | 7.92 | 36.65 | 1.28 | 54.02 |
| TZ_R | 0.01 | 11.26 | 26.01 | 1.13 | 61.59 |
| SQ_R | 0.10 | 8.44 | 36.01 | 1.17 | 54.27 |
| **Acidic_R** | 0.06 | 9.85 | 31.01 | 1.15 | 57.93 |
| TH_B | 0.16 | 13.21 | 12.30 | 3.25 | 71.08 |
| DM_B | 0.01 | 31.81 | 29.95 | 0.82 | 37.42 |
| **Alkaline_B** | 0.08 | 22.51 | 21.12 | 2.03 | 54.25 |
| TH_R | 0.11 | 5.91 | 25.31 | 1.51 | 67.15 |
| DM_R | 0.00 | 16.91 | 36.62 | 0.80 | 45.67 |
| **Alkaline_R** | 0.06 | 11.41 | 30.97 | 1.16 | 56.41 |

Table S3 Relative importance of each process in each bin of each sample across the four sites. Only the five most abundant bins are shown in this table. _B means bulk soil. _R means rhizosphere soil.

| Group | Index | Bin1 | Bin2 | Bin3 | Bin4 | Bin5 |
| --- | --- | --- | --- | --- | --- | --- |
| TZ_R | HeS | 0.000 | 0.000 | 0.000 | NaN | 0.000 |
| TZ_R | HoS | 0.000 | 0.000 | 0.000 | NaN | 0.000 |
| TZ_R | DL | 0.000 | 0.000 | 0.000 | NaN | 0.000 |
| TZ_R | HD | 0.000 | 0.265 | 0.000 | NaN | 1.000 |
| TZ_R | DR | 1.000 | 0.735 | 1.000 | NaN | 0.000 |
| TZ_R | DominantProcess | DR | DR | DR |  | HD |
| TZ_R | DominantProcessImportance | 1.000 | 0.735 | 1.000 | NaN | 1.000 |
| TZ_R | DominantProcessPvalue | 0.000 | 0.326 | 0.343 |  | 0.329 |
| TZ_B | HeS | 0.000 | 0.000 | 0.000 | 0.000 | 0.000 |
| TZ_B | HoS | 0.189 | 0.000 | 0.000 | 0.000 | 0.000 |
| TZ_B | DL | 0.000 | 0.000 | 0.000 | 0.000 | 0.000 |
| TZ_B | HD | 0.000 | 0.000 | 0.000 | 0.000 | 0.000 |
| TZ_B | DR | 0.811 | 1.000 | 1.000 | 1.000 | 1.000 |
| TZ_B | DominantProcess | DR | DR | DR | DR | DR |
| TZ_B | DominantProcessImportance | 0.811 | 1.000 | 1.000 | 1.000 | 1.000 |
| TZ_B | DominantProcessPvalue | 0.040 | 0.000 | 0.000 | 0.009 | 0.000 |
| Group | Index | Bin1 | Bin2 | Bin3 | Bin4 | Bin5 |
| DM_R | HeS | 0.000 | 0.000 | 0.000 | 0.000 | 0.000 |
| DM_R | HoS | 0.869 | 0.000 | 0.000 | 0.000 | 0.000 |
| DM_R | DL | 0.000 | 0.000 | 0.500 | 0.000 | 0.436 |
| DM_R | HD | 0.000 | 0.000 | 0.000 | 0.000 | 0.000 |
| DM_R | DR | 0.131 | 1.000 | 0.500 | 1.000 | 0.564 |
| DM_R | DominantProcess | HoS | DR | DL_DR | DR | DR |
| DM_R | DominantProcessImportance | 0.869 | 1.000 | 0.500 | 1.000 | 0.564 |
| DM_R | DominantProcessPvalue | 0.077 | 0.000 | 0.579_0.347 | 0.000 | 0.325 |
| DM_B | HeS | 0.000 | 0.000 | 0.000 | 0.000 | 0.000 |
| DM_B | HoS | 0.097 | 0.000 | 0.152 | 0.000 | 0.047 |
| DM_B | DL | 0.863 | 0.903 | 0.641 | 0.616 | 0.953 |
| DM_B | HD | 0.000 | 0.000 | 0.000 | 0.000 | 0.000 |
| DM_B | DR | 0.040 | 0.097 | 0.208 | 0.384 | 0.000 |
| DM_B | DominantProcess | DL | DL | DL | DL | DL |
| DM_B | DominantProcessImportance | 0.863 | 0.903 | 0.641 | 0.616 | 0.953 |
| DM_B | DominantProcessPvalue | 0.047 | 0.309 | 0.278 | 0.268 | 0.019 |
| Group | Index | Bin1 | Bin2 | Bin3 | Bin4 | Bin5 |
| SQ_R | HeS | 0.000 | 0.000 | 0.000 | 0.000 | 0.000 |
| SQ_R | HoS | 0.000 | 0.000 | 0.000 | 0.000 | 0.000 |
| SQ_R | DL | 0.781 | 0.380 | 0.990 | 0.900 | 0.807 |
| SQ_R | HD | 0.000 | 0.000 | 0.000 | 0.000 | 0.064 |
| SQ_R | DR | 0.219 | 0.620 | 0.010 | 0.100 | 0.128 |
| SQ_R | DominantProcess | DL | DR | DL | DL | DL |
| SQ_R | DominantProcessImportance | 0.781 | 0.620 | 0.990 | 0.900 | 0.807 |
| SQ_R | DominantProcessPvalue | 0.283 | 0.267 | 0.012 | 0.338 | 0.338 |
| SQ_B | HeS | 0.000 | 0.000 | 0.000 | 0.000 | 0.000 |
| SQ_B | HoS | 0.000 | 0.000 | 0.000 | 0.000 | 0.000 |
| SQ_B | DL | 0.671 | 0.000 | 0.680 | 0.127 | 0.649 |
| SQ_B | HD | 0.000 | 0.000 | 0.000 | 0.000 | 0.000 |
| SQ_B | DR | 0.329 | 1.000 | 0.320 | 0.873 | 0.351 |
| SQ_B | DominantProcess | DL | DR | DL | DR | DL |
| SQ_B | DominantProcessImportance | 0.671 | 1.000 | 0.680 | 0.873 | 0.649 |
| SQ_B | DominantProcessPvalue | 0.285 | 0.000 | 0.301 | 0.049 | 0.332 |
| Group | Index | Bin1 | Bin2 | Bin3 | Bin4 | Bin5 |
| TH_R | HeS | 0.000 | 0.000 | 0.000 | 0.000 | 0.000 |
| TH_R | HoS | 0.000 | 0.000 | 0.000 | 0.000 | 0.036 |
| TH_R | DL | 0.401 | 0.945 | 0.420 | 0.000 | 0.575 |
| TH_R | HD | 0.000 | 0.000 | 0.000 | 0.000 | 0.045 |
| TH_R | DR | 0.599 | 0.055 | 0.580 | 1.000 | 0.344 |
| TH_R | DominantProcess | DR | DL | DR | DR | DL |
| TH_R | DominantProcessImportance | 0.599 | 0.945 | 0.580 | 1.000 | 0.575 |
| TH_R | DominantProcessPvalue | 0.345 | 0.093 | 0.386 | 0.000 | 0.328 |
| TH_B | HeS | 0.000 | 0.000 | 0.000 | 0.000 | 0.000 |
| TH_B | HoS | 0.000 | 0.000 | 0.000 | 0.000 | 0.000 |
| TH_B | DL | 0.918 | 0.000 | 0.501 | 0.000 | 0.000 |
| TH_B | HD | 0.000 | 0.000 | 0.000 | 0.000 | 0.079 |
| TH_B | DR | 0.082 | 1.000 | 0.499 | 1.000 | 0.921 |
| TH_B | DominantProcess | DL | DR | DL | DR | DR |
| TH_B | DominantProcessImportance | 0.918 | 1.000 | 0.501 | 1.000 | 0.921 |
| TH_B | DominantProcessPvalue | 0.041 | 0.000 | 0.333 | 0.000 | 0.034 |
